# Critical Behavior and Macroscopic Phase Diagram of the Monoaxial Chiral Helimagnet Cr_1/3_NbS_2_

**DOI:** 10.1038/s41598-017-06728-5

**Published:** 2017-07-26

**Authors:** Eleanor M. Clements, Raja Das, Ling Li, Paula J. Lampen-Kelley, Manh-Huong Phan, Veerle Keppens, David Mandrus, Hariharan Srikanth

**Affiliations:** 1 Department of Physics, University of South Florida, Tampa, FL 33620 USA; 2 Department of Materials Science and Engineering, University of Tennessee, Knoxville, Tennessee 37996 USA

## Abstract

Cr_1/3_NbS_2_ is a unique example of a hexagonal chiral helimagnet with high crystalline anisotropy, and has generated growing interest for a possible magnetic field control of the incommensurate spin spiral. Here, we construct a comprehensive phase diagram based on detailed magnetization measurements of a high quality single crystal of Cr_1/3_NbS_2_ over three magnetic field regions. An analysis of the critical properties in the forced ferromagnetic region yields 3D Heisenberg exponents *β* = 0.3460 ± 0.040, *γ* = 1.344 ± 0.002, and *T*_C_ = 130.78 K ± 0.044, which are consistent with the localized nature the of Cr^3+^ moments and suggest short-range ferromagnetic interactions. We exploit the temperature and magnetic field dependence of magnetic entropy change (Δ*S*_M_) to accurately map the nonlinear crossover to the chiral soliton lattice regime from the chiral helimagnetic phase. Our observations in the low field region are consistent with the existence of chiral ordering in a temperature range above the Curie temperature, *T*_C_ < *T* < T*, where a first-order transition has been previously predicted. An analysis of the universal behavior of Δ*S*_M_(*T*,*H*) experimentally demonstrates for the first time the first-order nature of the onset of chiral ordering.

## Introduction

The chiral helimagnetic structures in noncentrosymmetric magnetic materials, which arise from the competition between the antisymmetric Dzyaloshinskii-Moriya interaction and symmetric exchange^1–3^, exhibit a range of variations in the nature of their magnetic ordering, such as itinerant vs. localized moments, critical behavior, and magnetocrystalline anisotropy. The cubic B20 helimagnets, MnSi^4, 5^, FeGe^6, 7^, and Fe_1−x_Co_x_Si^8, 9^ display itinerant magnetism, with the latter two belonging to the 3D Heisenberg universality class and the former exhibiting tricritical mean-field behavior. Cu_2_OSeO_3_, however, exhibits both localized ferromagnetism and belongs to the 3D Heisenberg class^10^. The weak anisotropy in these cubic systems allows the long-wavelength helimagnetic structure to be fixed along a single axis belonging to a set of equivalent crystallographic directions. In MnSi, the degeneracy of the <111> directions is lifted by a magnetic field. In FeGe, the preferred axes have a dependence on temperature. A common attribute of the phase diagrams of chiral helimagnets is a fluctuation-disordered precursor region above the magnetic ordering temperature which displays increasing chiral fluctuations as *T*_C_ is approached^10, 11^. The calculated *H-T* phase diagram of the chiral helimagnet Cr_1/3_NbS_2_ contains an analogous region above the Curie temperature, *T*_C_ < *T* < *T*_0_^12^. However, in this regime a *stable* chiral phase exists below a critical field.

Cr_1/3_NbS_2_ crystallizes in the noncentrosymmetric space group *P*6_3_22 with Cr atoms intercalated between planar 2H-type NbS_2_ layers^13–15^. Its unique magnetic properties arise from the strong uniaxial anisotropy of its hexagonal crystal structure paired with the localized nature of the Cr^3+^ moments. As a result, the chiral helimagnetic structure propagates along the c-axis^16, 17^. The large anisotropy does not allow the formation of the skyrmion lattice phase that is observed in the B20 helimagnets^18^. Instead, the application of a magnetic field *perpendicular* to the helical axis continuously transforms the spin chain into a chiral soliton lattice (CSL)^19^. In the CSL state, an applied magnetic field induces commensuration that competes with the symmetry protected chiral ordering to produce a modulated nonlinear magnetic state consisting of chains of ferromagnetic domains separated by 360° domain walls, called solitons. The spatial period of the CSL can be tuned with applied field and the macroscopic spin texture is robust against defects^19^. The CSL period diverges at a critical field, which drives a metamagnetic incommensurate to commensurate (IC-C) phase transition to a forced ferromagnetic (FFM) state.

Several studies have investigated the metamagnetic crossover from the chiral helimagnetic (CHM) phase into the CSL regime with experimental techniques such as bulk magnetization^20^, magnetoresistance^21^, and recently, AC susceptibility^22^. The phase boundaries in ref. 20 identified the magnetic field at which the magnetization reaches saturation as the critical field for the IC-C phase transition and the peak in the differential susceptibility, d*M*/d*H*, as the crossover from CHM to CSL. In ref. 22, analysis of the linear and nonlinear AC magnetic response, *M*_ω_ vs. *T*, in an applied dc field was used to identify two regions of the CSL: the low-field CSL-1, which displays a linear response to an AC magnetic field, and a higher-field highly nonlinear CSL-2, which shows a giant response in the third harmonic, *M*_3ω_. It was also suggested that the CHM state may exist only as a singularity at zero applied magnetic field. These features of the magnetic response were restricted to the phase boundaries between the high temperature paramagnetic state and the ordered phases below *T*_C_ in fixed dc fields. Thus, no signature of the dc-field-driven crossover from CSL-1 to CSL-2 could be directly observed.

The nature of the phase transition has been addressed both experimentally and theoretically via measurements of the specific heat and mean-field analysis, respectively. Heat capacity measurements have shown a lambda anomaly consistent with a second-order phase transition near *T*_C_^20^. However, using a mean-field approximation, Laliena *et al*. demonstrated that a first-order transition from the paramagnetic state to the CHM phase occurs at a zero-field critical temperature *T*_0_, above *T*_C_^12^. First-order transitions have been known to occur in the B20 chiral helimagnets and have been categorized as fluctuation-induced^23, 24^. The nature of the low field phase transition in Cr_1/3_NbS_2_ may be difficult to observe experimentally if it is weakly first-order, which could explain the lack of sharp divergence in the previous heat capacity measurements.

To shed light on the aforementioned issues, the magnetic transitions, critical behavior, and phase diagram of Cr_1/3_NbS_2_ have been investigated by DC magnetization, critical exponents analysis, and magnetic entropy change (Δ*S*_M_). Our combined analytical method has proven useful in uncovering the complex nature of magnetic multiphases and interactions, leading to establishment of the new comprehensive phase diagram of exotic systems such as the spin chain compound Ca_3_Co_2_O_6_^25^ and the multiferroic LuFe_2_O_4_^26^. In case of the monoaxial CHM, Cr_1/3_NbS_2_, we found that at magnetic fields above the critical field for the IC-C phase transition, a second-order transition to an FFM state occurs at *T*_C_. This transition is investigated via calculation of the critical exponents and is described by the 3D Heisenberg model. At moderate and low magnetic field, Δ*S*_M_ clearly defines the critical fields of the onset of the chiral and ferromagnetic phases, including crossovers within the CSL regime. These results confirm the existence of the linear CSL and the concurrent disappearance of the CHM phase for non-zero applied fields in a temperature region above and below *T*_C_. At lower temperatures, however, we demonstrate via magnetic entropy arguments that the chirality of the CHM state may remain completely preserved even at finite fields. Finally, we demonstrate for the first time a failure of the universality of Δ*S*_M_(*T*,*H*) that is consistent with the existence of first-order behavior at the phase transition in small magnetic fields.

## Background

### Critical Behavior

It is well known, according to Landau theory of second-order phase transitions^27, 28^, that the order parameter is small in the vicinity of the critical temperature. Thus the free energy can be expanded as a power function of the order parameter, *M*.

1 $${\rm{\Phi }}={{\rm{\Phi }}}_{0}+a/2\,{M}^{2}+b/4\,{M}^{4}+\mathrm{...}-HM$$

The linear term may be coupled to a field, *H*, if the ordered state involves a breaking of symmetry. The equilibrium condition is satisfied from minimizing the thermodynamic potential d*Φ*/d*M* = 0, leading to the equation of state that defines the behavior of the ordered state in the critical region. For a ferromagnetic system, the order parameter is simply the magnetization, i.e. the polarization. Therefore, the magnetic equation of state is

2 $$H=aM+b{M}^{3}.$$

In more exotic magnetic systems, such as the chiral helimagnet, the order parameter may be described by a slowly varying periodic spin density and may be multi-component, as in the B20 CHMs^23^. In Cr_1/3_NbS_2_, and other CHMs, a metamagnetic transition drives the system from the chiral state to a field-polarized ferromagnetic state. Thus, above a critical magnetic field, a thermally driven phase transition from the paramagnetic (PM) to FFM state can be described by a magnetic equation of state with order parameter *M*.

In the critical region near a second-order phase transition, the divergence of the correlation length, ${\rm{\xi }}={\xi }_{{0}}{|(T-{T}_{C})|}^{-{\rm{\nu }}}$, leads to a series of universal scaling laws. In the case of magnetization

3 $${M}_{S}\,(T)={M}_{0}\,{(-\varepsilon )}^{\beta },T < {T}_{C}$$

4 $${\chi }^{-1}(T)=(h/M){\varepsilon }^{\gamma },T > {T}_{C}$$

5 $$M=D{H}^{1/\delta },T={T}_{C}$$

where *M*_*0*_, *h/M*, and *D* are the critical amplitudes, respectively, of the spontaneous magnetization, the inverse susceptibility and the field dependence of the magnetization of the critical isotherm^29^. $\varepsilon =T-{T}_{C}/{T}_{C}$ is the reduced temperature. The critical exponents also may be calculated experimentally from magnetization measurements using the Arrott-Noakes equation of state^30^,

6 $${(H/M)}^{1/\gamma }=A\varepsilon +B{M}^{1/\beta }.$$

Therefore, the correct exponents are those by which *M* (*H*, *T*) is rescaled into a series of parallel lines, with the critical isotherm passing through the origin at *T* = *T*_C_ (hence *ε* = 0).

### Magnetocaloric Effect

The magnetocaloric effect (MCE) has been demonstrated to be an effective method to probe field- and temperature-dependent magnetic phase transitions^25, 26, 31–33^ and is evaluated via calculation of the magnetic entropy change, Δ*S*_M_. Isothermal magnetization versus applied magnetic field curves are measured with small steps for a range of temperatures near *T*_C_. By exploiting the thermodynamic Maxwell relation relating the change in magnetic entropy (*S*_M_) with respect to field to the change in *M* versus temperature

7 $${(\frac{\partial {S}_{M}(T,H)}{\partial H})}_{T}={(\frac{\partial M(T,H)}{\partial T})}_{H}$$

Δ*S*_M_ can be calculated by integrating between successive isotherms using the expression:

8 $${\rm{\Delta }}{{S}}_{{M}}={\int }_{{{H}}_{i}}^{{{H}}_{f}}{(\frac{\partial {M}(T,{H})}{\partial {T}})}_{{H}}d{H}$$

The sign of Δ*S*_M_ indicates the nature of the ordering of the magnetic state. In the conventional MCE, application of a magnetic field causes a decrease in magnetic entropy due to field-induced ordering of spins which suppresses thermal fluctuations, hence Δ*S*_M_ < 0. Conversely, application of a magnetic field may result in Δ*S*_M_ > 0. In the case of antiferromagnetic materials, the application of an external field causes spins to be rotated against their preferred direction in antiparallel sublattices. In general, a positive value of Δ*S*_M_ indicates a magnetic field-induced disorder with respect to the magnetic ground state, i.e. zero-field magnetic configuration. The information related to spin ordering obtained from MCE allows us to map out the phase evolution via conventional measurements as well as resolve details that have not been previously observed.

## Results and Discussion

### Magnetic Properties

**Figure 1:**
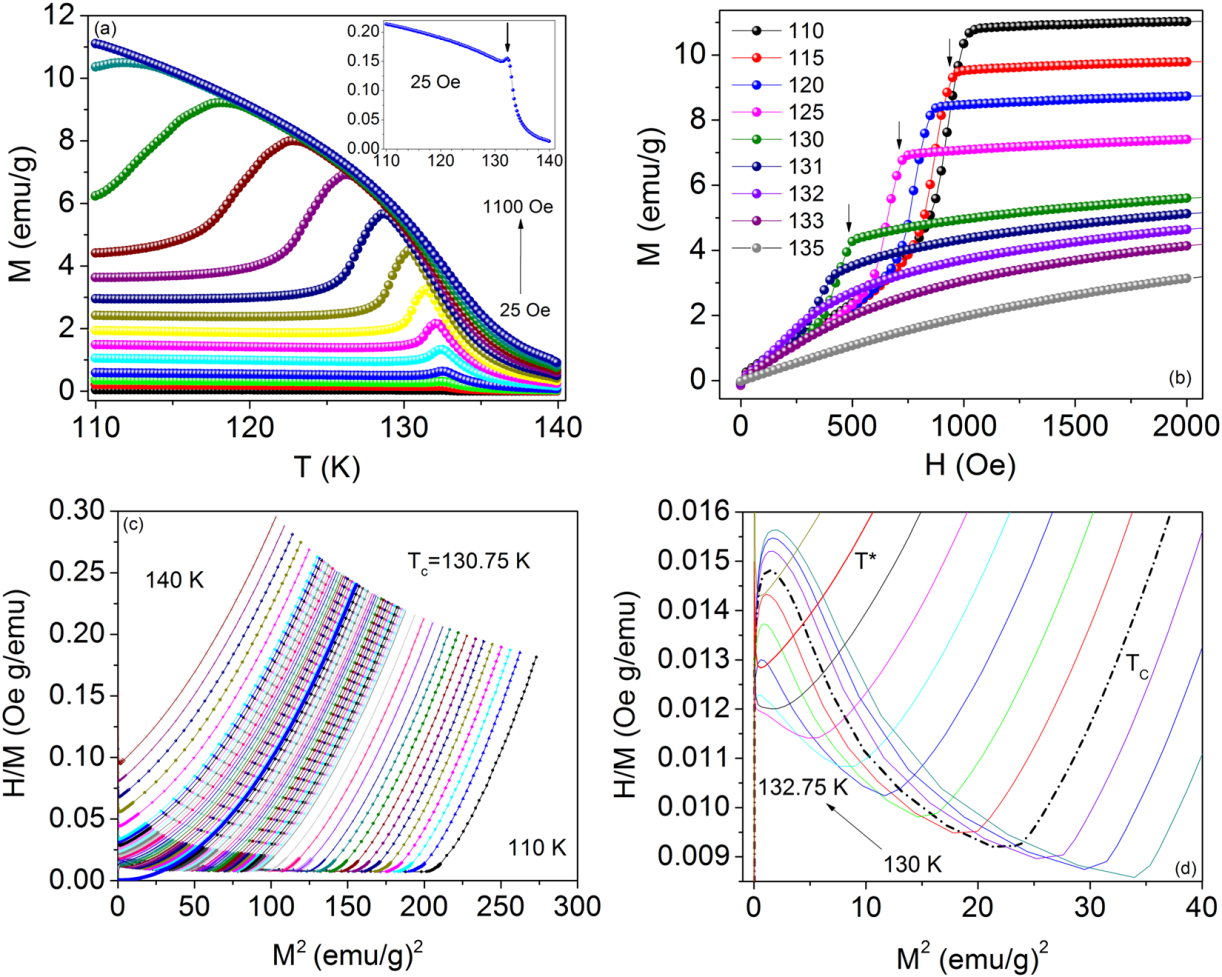
Temperature and field dependence of dc magnetization for *H*⊥*c*. (**a**) *M* vs. *T* for *H* = 25–1100 Oe. The inset shows the kink point associated with the onset of chiral ordering. (**b**) Zoomed view of *M* vs. *H* from *H* = 0–2 kOe. Arrows indicate the temperature dependence of the saturation field for the FFM state, *H*_FFM_(*T*). (**c**) *H/M* vs. *M* ^2^ for *H* = 0–30 kOe. The line represents a quadratic fit to the isotherm at 130.75 K, which defines *T*_C_. (**d**) Zoomed view of Arrott plot from *H* = 0–1 kOe. *H*_FFM_(*T*) occurs at the minimum of the negative slope region. The region shifts to successively smaller field ranges with increasing temperature.

Figure 1(a) shows the magnetization versus temperature for various applied magnetic fields in the easy plane, *H* ⊥ *c*, measured with a zero-field-cooled protocol (ZFC). As observed in previous studies, a sharp kink occurs at the onset of chiral ordering (inset), which broadens and shifts toward lower temperatures with an increase in applied magnetic field^14, 17, 34^. Similar behavior exists in the cubic chiral helimagnets where the inflection point marks the onset of a fluctuation-disordered precursor region that precedes chiral magnetic ordering at the kink point^4–10^. At low applied fields, *H* = 50–225 Oe, the kink occurs at a constant temperature, *T* = 132 K. At *H* = 425 Oe, the peak occurs at *T* = 130.75 K.

Magnetization versus magnetic field applied perpendicular to the *c* axis is shown in Fig. 1(b). Three distinct regions appear in *M* vs. *H* below *T*_C_ - the low field linear region, the sharp nonlinear increase in *M* in the CSL state at intermediate *H*, and saturation at *H*_FFM_(*T*), the critical field corresponding to the FFM phase^14, 16, 17, 20^. At 110 K, the measured saturation field is 1 kOe. At higher temperatures, the field required for the onset of the FFM state continuously drops to lower values, as indicated by the arrows in Fig. 1(b).

Figure 1(c) shows the (inverted) Arrott plot, *H/M* vs. *M* ^2^, for *T* = 110–140 K. The upward curvature clearly indicates that the ferromagnetic interactions cannot be described as mean field, i.e. *β* = 0.5 and *γ* = 1 in the Arrott-Noakes equation. A quadratic extrapolation^35^ to zero field, performed for the field range *H* = 1–30 kOe, gives *T*_C_ = 130.75 K. Figure 1(d) shows negative slopes in the Arrott isotherms below the saturation field *H*_FFM_(*T*) for temperatures near *T*_C_. Negative slope behavior exists for isotherms measured from *T* = 110–132 K and is likely due the nature of the CSL. In this region, an applied magnetic field induces jumps^36^ in the soliton lattice period (ferromagnetic domains) causing a rapid increase in *M*. As the magnetization in the CSL increases faster than the field, a negative slope occurs in (*H*/*M*)vs. *M* ^2^. Thus, we stress that the negative slope behavior should not be interpreted as satisfying the Banerjee criterion^37^, *b* < 0 in equation (2), which is commonly used to identify a first-order transition within Landau phenomenology. In terms of the field-driven transition, the change in period of the CSL with applied field is a *continuous* process^19^. The CSL has also been noted to have irreversible behavior in *M* vs. *H* that could be mistaken as a first-order phenomenon, namely hysteresis upon cycling the field up and down^20^. However, this is likely due to different energy barriers for the exit and entry of solitons as the field is cycled through saturation magnetization.

### Critical Exponents Analysis

For fields exceeding *H*_FFM_ (*T*) it can be seen that the slopes of the Arrott plots are positive-only, consistent with a second-order phase transition. To confirm the nature of the paramagnetic to FFM phase transition and to verify the correct value of *T*_C_, critical exponents were calculated for *H* = 1–30 kOe. The field range for the analysis is restricted to the FFM region of the phase diagram, which ensures the validity of the magnetic equation of state.

An iterative procedure using the Kouvel-Fisher method^38^ generates values for *T*_C_, *β*, and *γ*, which are subsequently fitted to the Arrott-Noakes equation until the critical values converge. In this analysis, Eqs (3) and (4) are re-written in the form:

9 $${M}_{S}(T){[{\rm{d}}{M}_{S}(T)/{\rm{dT}}]}^{-{\rm{1}}}=(T-{T}_{c})/\beta \,$$

10 $${\chi }_{0}^{-1}(T){[{\rm{d}}{\chi }_{0}^{-1}(T)/{\rm{dT}}]}^{-{\rm{1}}}=(T-{T}_{c})/\gamma .\,$$

**Figure 2:**
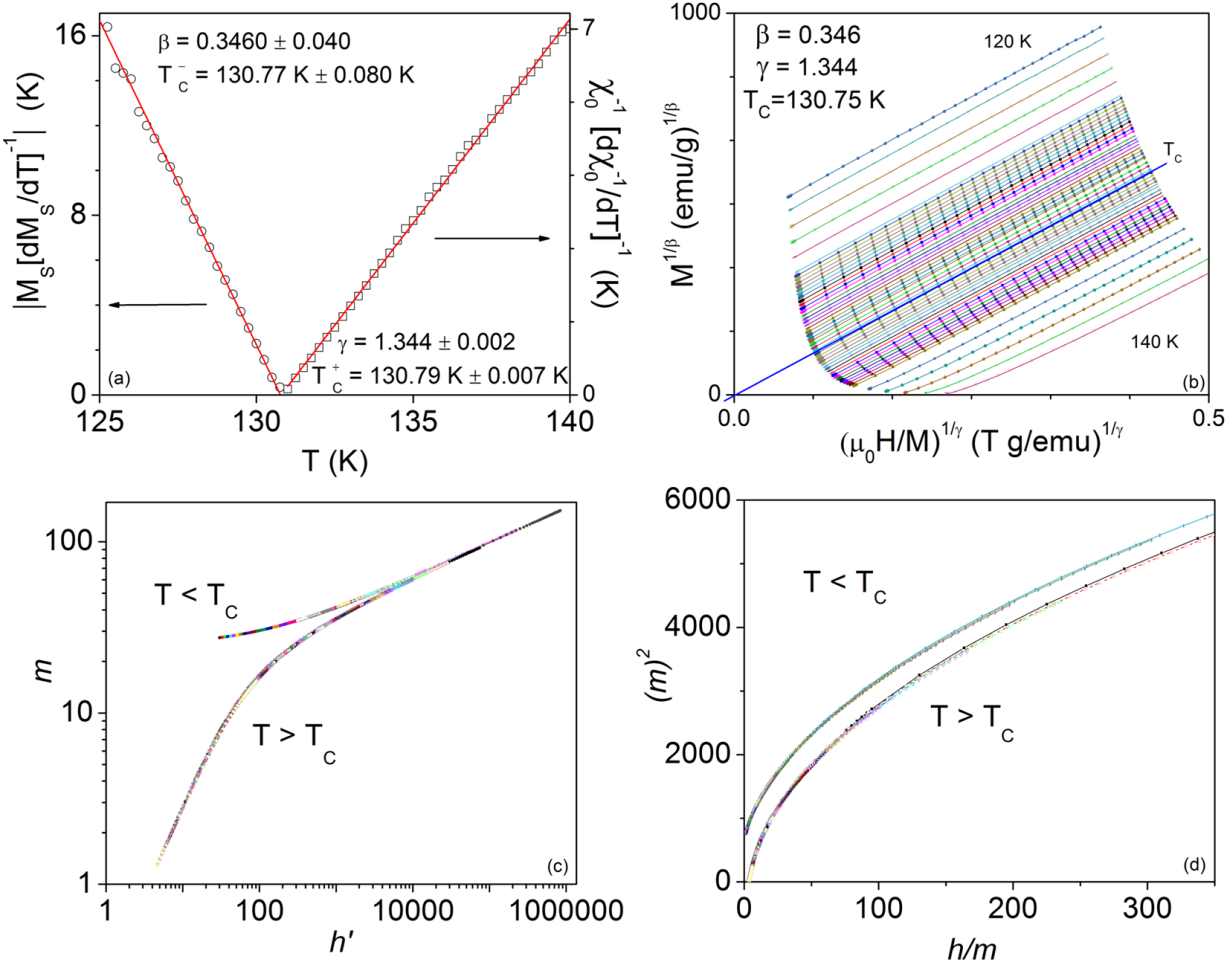
(**a**) Kouvel-Fisher plots from the reformulated spontaneous magnetization and initial inverse susceptibility data. Linear fits yield *β*, *γ*, *T*_C_^+^, and *T*_C_^−^. (**b**) Modified Arrott plot with the obtained critical exponents, which fall into the 3D Heisenberg class. A linear fit passing through the origin confirms *T*_C_ = 130.75 K. (**c**) Renormalized magnetization isotherms according to the equations of state from (11) and (**d**) (12) which confirm universal behavior.

Plots of ${M}_{{\rm{s}}}(T){[{\rm{d}}{M}_{{\rm{s}}}(T)/{\rm{d}}T]}^{-1}$ vs. *T* and ${\chi }_{0}^{-1}(T){[{\rm{d}}{\chi }_{0}^{-1}(T)/{\rm{d}}T]}^{-1}$ vs. *T* result in straight lines with slopes of 1/*β* and 1/*γ* respectively, which intercept the temperature axis at *T*_C_ (Fig. 2(a)). This procedure yields the critical exponents *β* = 0.3460 ± 0.040 *γ* = 1.344 ± 0.002 and *T*_C_ = 130.78 K ± 0.044. These critical exponents are used to construct the modified Arrott plot (Fig. 2(b)). The line represents a linear fit to the isotherm at 130.75 K.

To test the validity of the calculated exponents, the critical isotherms are also rescaled according to the renormalized magnetic equations of state

11 $$m={f}_{\pm }(h)$$

12 $$h/m=\pm {a}_{\pm }+{b}_{\pm }{m}^{2}$$

where $m\equiv |\varepsilon {|}^{-\beta }M(H,\varepsilon ),$ $h\equiv |\varepsilon {|}^{-\beta \delta }H$ are the renormalized magnetization and field^39, 40^, respectively. If the correct values for the critical exponents and *T*_C_ are used, the data should collapse onto universal curves above and below *T*_C_, signified by *f*_±_ in equation (11). As shown in Fig. 2(c) and (d), the data collapse well, indicating the validity of the above analysis. This confirms the second-order picture of the PM-FFM phase transition, as well as the correctness of the exponents. Our results agree with the specific heat results in ref. 20.

We note that the critical exponent values of Cr_1/3_NbS_2_ (*β* = 0.3460 ± 0.040, *γ* = 1.344 ± 0.002) match well with those of the 3D Heisenberg model (*β* = 0.365 ± 0.003, *γ* = 1.386 ± 0.004). The 3D Heisenberg-like ferromagnetism appears to be appropriate for the localized nature of the Cr^3+^ moments (*S* = 3/2), which have been reported to have a moment that saturates at ~3 μ_B_/Cr^20^. Although the model implies short-range interactions, the low-field helimagnetic structure shows a robust spin coherence, which suggests a long-range order that is set by the underlying crystalline chirality^19, 41^. Thus, saturating the system to the FFM state decouples the competing symmetric and DM interactions, and reveals the principal magnetic ordering to be that of short-range interactions, a signature of the strong ferromagnetic exchange component of the system. In a report by Dyadkin *et al*.^42^, 3D Heisenberg exponents were calculated for a reduced-symmetry P6_3_ polytype of Cr_1/3_NbS_2_ with disorder of Cr ions among three independent lattice positions, which showed no signatures of chiral magnetism and only ferromagnetic ordering below *T*_C_ = 88 K for all field ranges. The lack of helical ordering suggests a breakdown of necessary noncentrosymmetry in the Cr sublattice despite the chiral nature of the NbS_2_ layers^3^. Thus our critical exponents, which describe a Heisenberg-like ferromagnetic subsystem, are consistent with the system that lacks chiral ordering but preserves the symmetric exchange.

It has been theoretically shown^12^, through a mean-field approximation, that a second-order phase line in Cr_1/3_NbS_2_ is terminated by a tricritical point below which a first-order transition appears in a region *T*_C_ < *T* < T*. In the following section, the magnetic entropy change will be analyzed to define the boundaries in the chiral phase as well as determine a value for T*. The magnetic entropy change results will also be used to test the universality in different regions of the phase diagram to identify a possible first-order transition.

### Magnetic Entropy Change: Temperature and Field Dependent Phase Boundaries

**Figure 3:**
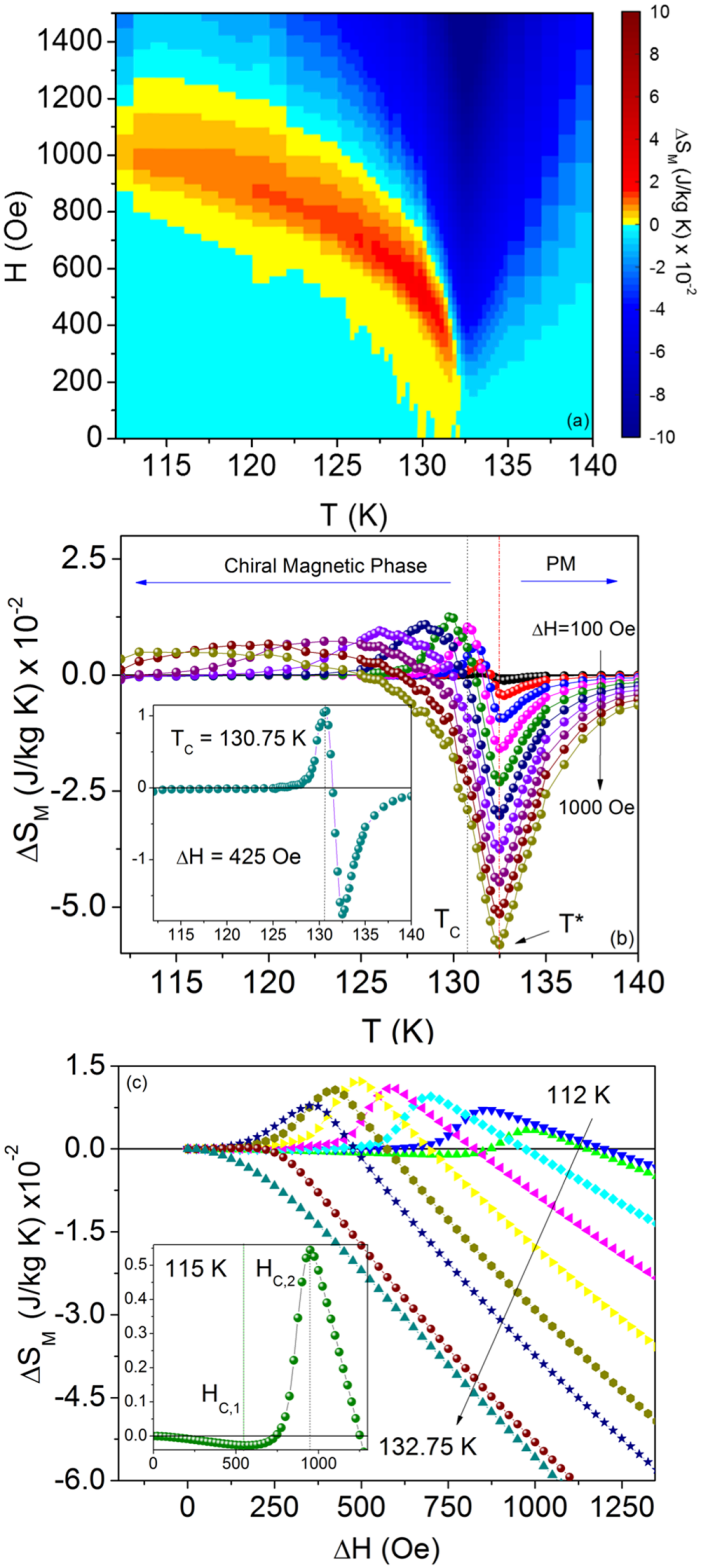
Magnetic entropy change as a function of temperature and field. (**a**) *H* − *T* surface plot of Δ*S*_M_. (**b**) Δ*S*_M_ vs. *T* for Δ*H* = 100–1,000 Oe, which shows the behavior of the chiral and PM phases. For clarity, Δ*S*_M_(*T*) is shown in steps of Δ*H* = 100 Oe. A temperature gap, Δ*T*, exists between *T*_C_ and the order-disorder transition at T*. Inset: Δ*S*_M_ vs. *T* for Δ*H* = *H*_C_(*T*_C_). (**c**) Δ*S*_M_ vs. Δ*H* for temperatures below and above T*. Inset: Δ*S*_M_ at 115 K which shows the peak, *H*_C,2_, above which Δ*S*_M_ monotonically decreases with *H*, defined as the IC-C transition. The local minimum at *H*_C,1_ defines the CHM−CSL crossover.

The magnetic entropy change surface plot (Fig. 3(a)) depicts the general behavior of the temperature and field dependence of the phase boundaries. Regions of positive and negative Δ*S*_M_ are represented with warm and cool colors, respectively. This graph has similar behavior to reported phase diagrams^12, 20–22^, namely the gradual decrease in critical field, *H*_FFM_ (*T*), with increasing temperature. It suggests that thermal fluctuations play an important role in the stability of the CSL^43^. To resolve the details of the entropy surface plot, Δ*S*_M_ vs. *T* and Δ*S*_M_ vs. Δ*H* are analyzed separately in Fig. 3(b and c), respectively.

Figure 3(b) shows the temperature dependence of Δ*S*_M_ for Δ*H* = 100–1,000 Oe, spanning the chiral phase. In the paramagnetic region, finite values of Δ*S*_M_ persist up to 140 K, well above *T*_C_, which suggests that ferromagnetic correlations may be present even at higher temperature. The most prominent feature in Δ*S*_M_(*T*) is the field-independent, global minimum at *T* ~ 132.5 K, above the Curie temperature of 130.75 K determined in the previous section. Given its relation to the derivative of the magnetization, ∂M/∂T, the behavior of the magnetic entropy change at the global minimum indicates an order-disorder transition^44^ at T* ~ 132.5 K ± 0.13 K. In the cubic chiral helimagnets, an inflection point in *M* vs. T marks the onset of a precursor region of increasing chiral correlations which precedes the transition to the chiral magnetic phase at *T*_C_. However according to theoretical results, Cr_1/3_NbS_2_ exibits a stable chiral phase within this temperature gap region, Δ*T*, which indicates a phase transition at T*. Evidence of this ordering in Δ*T* can be observed by the variation in the location of Δ*S*_M,*max*_(*T*) between each field change. The inset shows a representative curve for Δ*H* = 425 Oe, where the positive peak in Δ*S*_M_ occurs at *T* = *T*_C_. For Δ*H* < 425 Oe, Δ*S*_M,*max*_ occurs at successively higher temperatures between *T*_C_ < *T* < T*. *H*_C_ (*T*_C_) = 425 Oe is thus defined as the critical field below which the CSL exists above *T*_C_.

**Figure 4:**
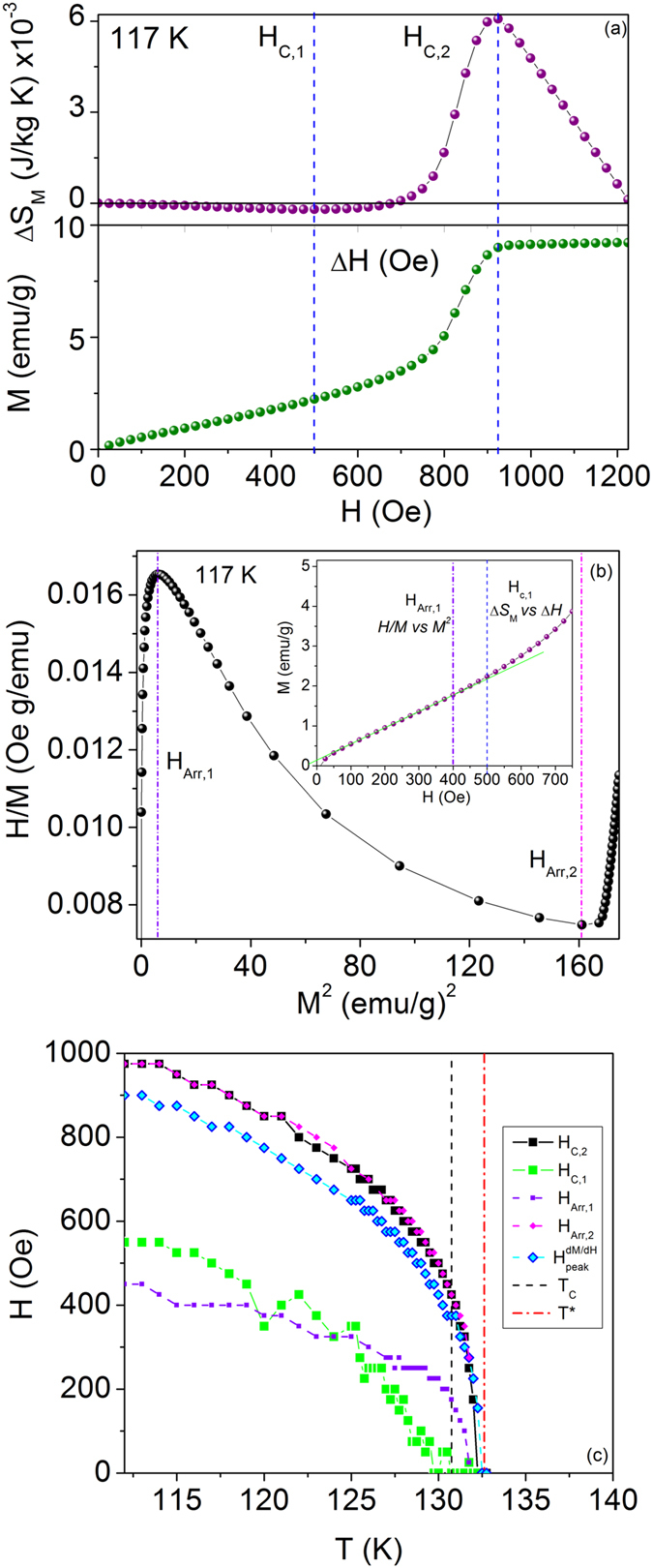
(**a**) Arrott plot and (inset) *M* vs. *H* at 115 K. The lower and upper field limits of the negative slope region are defined as *H*_Arr,1_ and *H*_Arr,2_. *H*_Arr,1_ occurs at fields where *M* vs. *H* deviates from linearity and is compared to *H*_C,1_. *H*_Arr,2_ occurs at the forced ferromagnetic transition. (**b**) *H*-*T* phase diagram defined by the characteristic fields.

The metamagnetic crossover and IC-C phase transition boundaries are clearest by examining the field dependence of Δ*S*_M_ (Fig. 3(c)). For temperatures ranging from 110–129.5 K, Δ*S*_M_ in the low Δ*H* regime linearly decreases with applied field. This can be seen in the inset of Fig. 3(c), which shows Δ*S*_M_ vs. Δ*H* for 115 K. Upon reaching a local minimum, the entropy of the spin system begins to rise at a critical field *H*_C,1_. Δ*S*_M_ reaches a maximum at *H*_C,2_ above which the entropy monotonically decreases with increasing field, characteristic of a ferromagnetic state. Figure 4(a) compares the critical fields derived from Δ*S*_M_ vs. Δ*H* to points along *M* vs. *H*. *H*_C,2_ clearly corresponds to *H*_FFM_, the critical field for the IC-C phase transition to the FFM state.

Below *H*_C,2_, the chiral phase is divided into two regions of opposite sign of Δ*S*_M_. *H*_C,1_ defines a crossover field in the chiral phase. To interpret this crossover, it is necessary to consider the balance of energies that lead to the stabilization of the CSL. It is underlined by Kishine and Ovchinnikov^41^ that the chiral helimagnetic ground state is forced to break chiral symmetry and is thus protected by the underlying crystalline chirality. When a magnetic field is applied perpendicular to the helical axis, the field-induced tendency towards commensuration competes with the protected chirality, eventually causing the CHM-CSL crossover. The applied magnetic field clearly disorders the chiral helimagnetic ground state and it is thus reasonable to define the crossover from CHM to CSL from the critical field at which Δ*S*_M_ begins to increase, *H*_C,1_. As the CSL period increases and the commensurate domains grow with applied field, Δ*S*_M_ continues to increase until the IC-C phase transition at *H*_C,2_. The boundaries defined by *H*_C,2_ and *H*_C,1_ are plotted in Fig. 4(c). For ~129.75 K ≤ *T* < T*, entropy values are only positive in the chiral phase, i.e. *H*_C,1_ drops to 0 Oe and no pure CHM phase exists for non-zero field. This behavior agrees well with the results in refs 12 and 22, which demonstrate the existence of a PM-CSL phase transition at non-zero dc magnetic field.

The deviation of *M* vs. *H* curves from linearity have been noted as the possible boundary between the linear CSL-1 and nonlinear CSL-2 states^22^. As discussed previously, the negative slope region of *H/M* vs. *M* ^2^ is attributed to the rapid increase in the magnetization that occurs as the period of the CSL grows with increasing magnetic field. Figure 4(b) shows an (inverted) Arrott isotherm at 117 K with the lower and upper magnetic field boundaries of the negative slope region labelled by *H*_Arr,1_ and *H*_Arr,2_, respectively. *H*_Arr,2_ is found to correspond exactly with *H*_C,2_ (Fig. 4(c)). *H*_Arr,1_, however, deviates from *H*_C,1_, with *H*_Arr,1_ < *H*_C,1_ from 112 K until a crossover at ~125.5 K. The locations of *H*_Arr,1_ and *H*_C,1_ are compared to *M* vs. *H*, as shown in the inset of Fig. 4(b). For all temperatures measured, *H*_Arr,1_ was found to agree well with the deviation from linearity of the *M* vs. *H* curves. To confirm the location, linear fits were done for a range of field points for which the *R* ^2^ ≥ 0.99990 and the chi-squared ≤ 5.00 × 10^−6^. Above 125.5 K, *H*_C,1_ descends toward 0 Oe and falls below *H*_Arr,1_. This reveals a region which displays both increasing magnetic entropy and linearity of *M* vs. *H*. Based on the present results and the results in ref. 22, we define this region as the linear CSL regime.

The transition from the CHM to CSL regime is a nonlinear crossover within the same modulated phase^20^ and is distinct from a true phase transition. Therefore, it may lack a clear anomaly in experimental measurements. However, there are distinctly different behaviors below and above *H*_C,1_ and *H*_Arr,1_ in the magnetic entropy change and the Arrott plot, respectively. The field-dependent CHM-CSL and CSL-1–CSL-2 boundaries may have been impossible to observe with temperature-dependent AC magnetic response in ref. 22. We define the CHM phase as the region bounded from above by *H*_C,1_ and *H*_Arr,1_ in which ΔS_M_ is decreasing and *M* vs. *H* changes linearly. The variation between *H*_C,1_ and *H*_Arr,1_ below 125.5 K may be a result of the nonlinear crossover process between the CHM phase and the CSL regime.

The characteristic fields are plotted in Fig. 4(c) and show the phase line for the IC-C transition and the region marking the nonlinear crossover from CHM to CSL. *H*_C,2_ persists past *T*_C_ dropping to zero near T*. The IC-C phase line in the temperature range *T*_C_ − T* is consistent with the theoretically reported phase diagram^12^ in which the chiral phase is stable above *T*_C_. This also agrees well with magnetoresistance results in ref. 21 in which a sharp peak and broad shoulder correspond to two isothermal lines near *T*_C_ in the reported phase diagram.

The CSL-FFM crossover field identified from conventional magnetization measurements as the peak in the differential susceptibility (d*M*/d*H*), *H*_peak_, lies within the CSL regime defined by *H*_C,1_ and *H*_C,2_ (Fig. 4(c)). The narrow extent of the region between *H*_peak_ and *H*_C,2_ resembles the highly nonlinear CSL region obtained in the theoretical phase diagram reported in ref. 12. The maximum values of entropy change (dark red region in Fig. 3(a)) are observed in the highly nonlinear CSL regime between approximately 125 K and 131.5 K, where crossing of energy levels leading to the increase in CSL period occurs rapidly, causing sharp increases in magnetization^45^.

We recall that below a critical magnetic field the IC-C phase line has been predicted^12^ to mark a first-order transition from the PM state into the CSL state. Magnetic entropy change results can be used to determine the order of the transition based on the existence or failure of the universal behavior of Δ*S*_M_ expected for a second-order phase transition, as presented in the following section.

### Universal Behavior

The scaling of Δ*S*_M_(*T*) curves in the vicinity of a second-order phase transition has been theoretically grounded^46, 47^ and experimentally confirmed^33, 48, 49^ in a variety of magnetic systems based on the power law dependence of Δ*S*_M_ ∝ *H* ^n^. Thus, equivalent points around the transition temperature of Δ*S*_M_(*T*) curves measured up to different maximum applied fields (Δ*H*) should collapse onto the same point of the universal curve when properly rescaled. The universal curve for magnetic entropy change can be constructed by normalizing Δ*S*_M_(*T*) curves by the maximum value of |Δ*S*_M_^peak^|, which occur at the transition temperature, *T*_peak_^44^. The temperature axis is rescaled with respect to a reference temperature such that Δ*S*_M_(*T*_r_)/Δ*S*_M_(*T*_peak_) ≥ 0.5. However, two reference temperatures, *T*_r1_ > *T*_peak_ and *T*_r2_ < *T*_peak_, are typically chosen, as will be discussed below. The transition of interest is that occuring at T* ~ 132.5 K. The references were chosen such that Δ*S*_M_(*T*_r1_)/Δ*S*_M_(*T*_peak_) = Δ*S*_M_(*T*_r2_)/Δ*S*_M_(*T*_peak_) = 0.75. The rescaled temperature axis is defined as

13 $$\theta =\{\begin{array}{cc}-(T-{T}_{c})/({T}_{r1}-{T}_{c}), & T\le {T}_{c}\\ (T-{T}_{c})/({T}_{r2}-{T}_{c}), & T > {T}_{c}\end{array}$$

such that *θ* = −1 for *T* = *T*_r1_.

**Figure 5:**
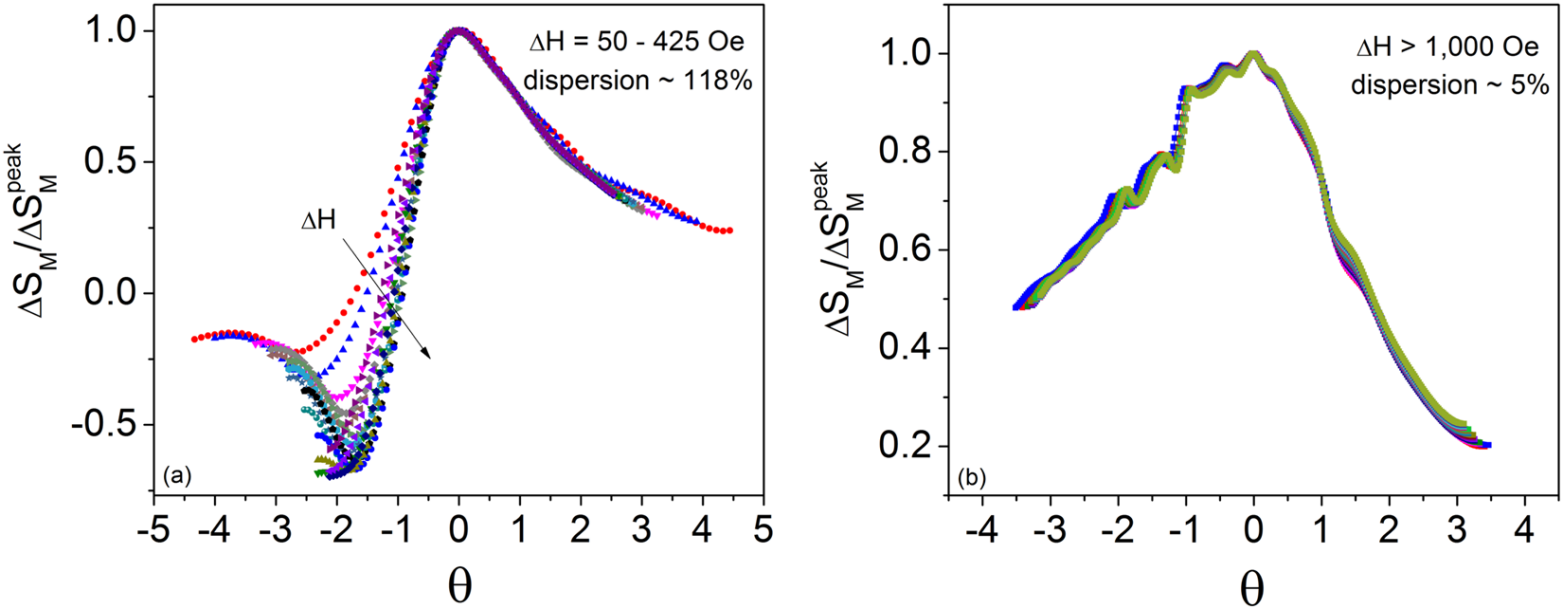
Rescaled Δ*S*_M_ vs. *T* curves for (**a**) Δ*H* = 25 Oe–425 Oe. The dispersion decreases with Δ*H* indicating first-order behavior that is suppressed to second-order with increasing field. (**b**) Universal curves for Δ*S*_M_ calculated only for fields above 1 kOe. Collapse indicates second-order behavior.

Figure 5(a) shows the rescaled curve constructed in the region *T*_C_ ≤ *T* ≤ T* for Δ*H* = 50–425 Oe. A second universal curve is constructed for applied fields in the FFM region in Fig. 5(b). To remove contributions from the low field phase that may have first-order behavior, Δ*S*_M_(*T*) was recalculated by changing the limits of integration in (1) to *H*_i_ = 1 kOe Oe and *H*_f_ = 30 kOe. The data near the PM-FFM transition scale well onto a universal curve with a disperson of only ~5% for a reference *θ* = −2^49^. The behavior in Fig. 5(b) agrees with the second-order nature that was established previously via the renormalized equation of state depicted in Fig. 2(c and d).

The rescaled Δ*S*_M_(*T*) curves in Fig. 5(a) do not collapse onto a universal curve and show a much higher degree of dispersion (~118%) below *T*_*peak*_. The failure of collapse of Δ*S*_M_(*T*) has been well-studied in a wide variety of compounds^49–51^. In certain systems, the lack of scaling of the magnetic entropy change has been attributed an additional magnetic phase which has increasing fluctuations near the magnetic ordering temperature at *T*_peak_^50^. However, the use of 2 reference temperatures corrects the failure and collapse can still be achieved if the transition is indeed second-order. However, if collapse continues to fail, extra entropy from a coexisting magnetic phase can be ruled out and the dispersion signifies a first-order transition^49, 50^. The dispersion in Δ*S*_M_/Δ*S*_M_^peak^(*θ*) typically exceeds 100% in magnetic systems with a first-order transtion^49^. This effect has been demonstrated in a wide variety of compounds and has even been successful in identifying the *weakly* first-order transition in DyCo_2_^49^. In Cr_1/3_NbS_2_, the IC-C second-order phase line is predicted^12^ to be terminated by a tricritical point at a critical magnetic field below which a first-order transition occurs from PM-CSL. The large dispersion shown in Fig. 5(a) gradually reduces with higher applied magnetic fields. This may be a signature of the suppression of the first-order character to second-order. The suppression of first- to second-order with magnetic field has also been shown to occur in the cubic chiral helimagnets. The present results within the Δ*S*_M_(*T*) scaling model are entirely consistent with the first-order transition that has been theoretically predicted for Cr_1/3_NbS_2_^12^.

In the chiral helimagnets, first-order transitions have been identified as occuring through a fluctuation-induced discontinuous transition^23, 24^. Such transitions exist in systems that would otherwise be second-order as defined by the symmetry conditions of Landau theory^52^. Therefore as the system approaches the phase transition, an excess of critical fluctuations causes the order parameter to evade the critical point^24^. A mechanism proposed by Bak and Jensen for MnSi and FeGe is a first-order transition that may be driven by the self-interaction of an order parameter with a large number of components - defined with respect to cubic symmetries^23^. However, Janoschek *et al*. demonstrated experimentally that critical helimagnetic fluctuations are driven first-order on the length scale of the DM interaction for MnSi, before the weak cubic anisotropies take effect^23, 24^. A recent study on Cu_2_OSeO_3_ suggests that strong fluctuations arise on a length scale above the DM interaction^10^. Interestingly, 3D Heisenberg critical exponents were calculated for Cu_2_OSeO_3_, while MnSi displays tricritical mean-field behavior^5, 10^. In these cubic systems, the hierarchy of energy scales go as ${\rm{J}}\gg {\rm{DM}}\gg {{\rm{A}}}_{{\rm{cub}}}$, or equivalently length scales ${{\rm{\xi }}}_{{\rm{FM}}}\ll {{\rm{\xi }}}_{{\rm{DM}}}\ll {{\rm{\xi }}}_{{\rm{cub}}}$ ^24^. In Cr_1/3_NbS_2_, however, the large single-ion anisotropy could lead to a different hierarchy. Calculations in ref. 53, give *J*_⊥_ = 1.4 × 10^2^ K, *J*_||_ = 15 K, and *D* = 2.9 K, which satisfies the requirement *D*/*J*_||_ = 0.16 and demonstrates the strong FM interactions in the a-b plane, *J*_⊥_, that give a relatively high *T*_c_. Another study^54^ gives the ratio of the anisotropy energy to the exchange energy as *A*/*J*_⊥_ = 0.10. Using these values, we estimate *A* = 14 K thus suggesting the length scales ${{\rm{\xi }}}_{{\rm{FM}}}\ll {{\rm{\xi }}}_{\mathrm{single}-\mathrm{ion}}\ll {{\rm{\xi }}}_{{\rm{DM}}}$. However, to determine the strength of the interactions that may cause the first-order transition, the Ginzburg scale *ξ*_G_, the length scale at which fluctuations become strongly interacting, would need to be calculated. In Cu_2_OSeO_3_, *ξ*_G_ is found to be above *ξ*_DM_, which implies that strong interactions of the fluctuations would occur at energies above the DM interaction scale. This method could be applied in a future work for Cr_1/3_NbS_2_.

### Phase Diagram

**Figure 6:**
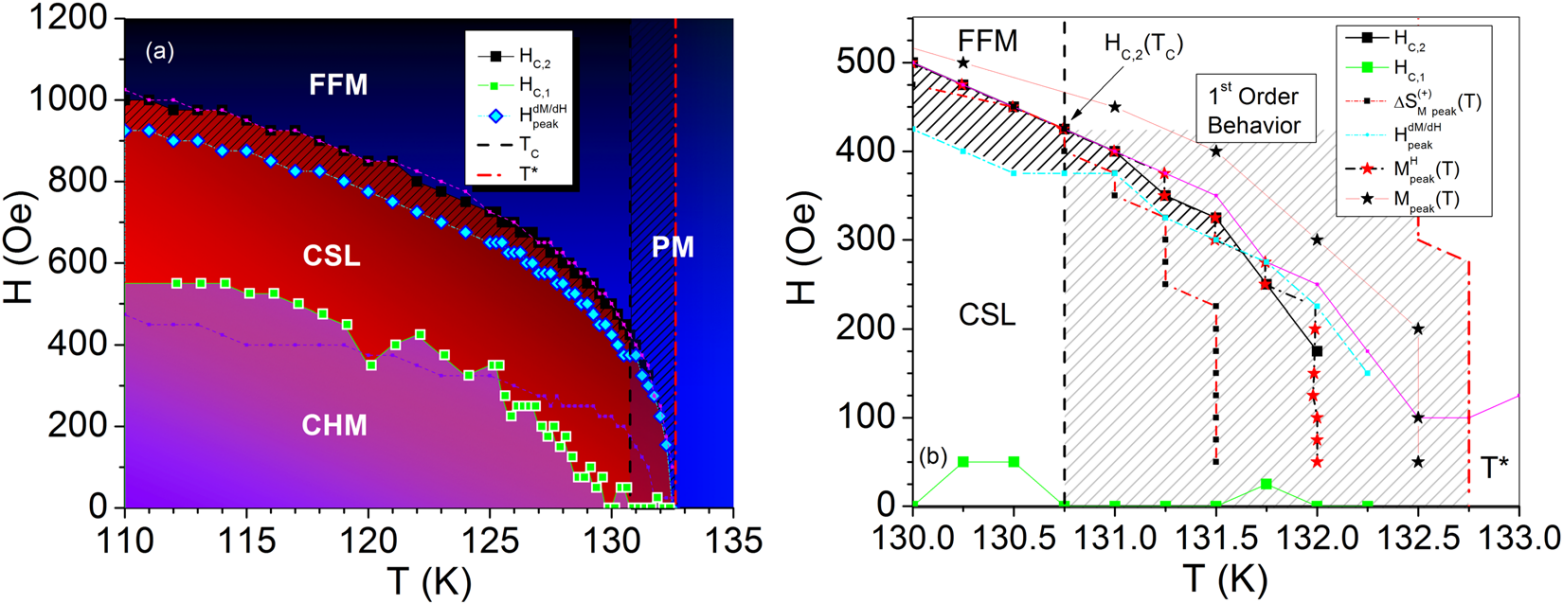
*H*-*T* phase diagram from Δ*S*_M_(*T*,*H*) and magnetization data. (**a**) Δ*T* is indicated by the shaded region between *T*_C_ and T*. The hashed area between *H*_C,2_ and *H*_peak_ defines the highly nonlinear CSL. The nonlinear CSL is bounded from above by *H*_peak_ and from below by *H*_C,1_ and *H*_Arr,1_. *H*_C,1_ and *H*_Arr,1_ (purple line) form a pocket within the chiral phase of the linear CSL. *H*_Arr,2_ (pink line) corresponds exactly to *H*_C,2_. (**b**) *H*-*T* phase diagram in the Δ*T* region where first-order behavior exists. The phase line for onset of CSL is a steep boundary at 132 K, indicated by red stars, where a first-order transition may exist. Irreversibility in this region can be seen by comparing *M* vs. *T* peaks measured with a ZFC protocol (black stars) and *M* vs. *T* peaks reformulated from *M* vs. *H*:*M* ^H^(*T*) (red stars).

A comprehensive phase diagram is shown in Fig. 6(a) with phase lines and crossover boundaries determined from Δ*S*_M_ and *H/M* vs. *M* ^2^. The shading separates regions of relative increase and decrease in Δ*S*_M_. The CSL, which corresponds to the region of increasing Δ*S*_M_ (shown in red), consists of three apparent regimes, the linear region between *H*_C,1_ and *H*_Arr,1_, nonlinear region between *H*_Arr,1_ and *H*_peak_, and highly nonlinear region between *H*_peak_ and *H*_C,2_. The hashed area between the FFM phase line and the d*M*/d*H* peak indicates where the highly nonlinear CSL may exist. Chiral ordering exists at applied fields below *H*_C,2_ in the temperature gap region, Δ*T*, between *T*_C_ = 130.75 K and T* = 132.5 K. At magnetic fields greater than *H*_C_(*T*_C_) = 425 Oe, indicated by a hashed area in Δ*T*, chiral fluctations are suppressed and PM-FM transition occurs at *T*_C_.

Figure 6(b) shows the phase diagram in Δ*T* = *T*_C_ − T*. The light gray shaded region indicates the first-order regime characterized by non-universal behavior of the rescaled magnetic entropy change. *H*_C,2_ is 425 Oe at *T*_C_ and persists until 132 K at a value of 175 Oe. At this temperature, the phase line separating CSL from PM drops off sharply and Δ*S*_M_ vs. *T* crosses zero for fields below 225 Oe. The sharpness of this drop off has been observed previously and was noted to resemble the sharpness of the MnSi first-order phase line^5, 22^. Evidence of irreversibility in Δ*T* can be seen from the 0.5 K offset (well within the resolution of our instrument) of the *T*_C_(*H*) lines determined from the kink points in *M* vs. *T* curves collected with a ZFC protocol (black stars) or reconstructed from *M* vs. *H* data (red stars). The highly nonlinear CSL bounded by *H*_C,2_ and *H*_peak_ is indicated by the dark hashed region. Resolution of the measurements do not allow the exact determination of the possible tri-critical point, however the convergence of *H*_C,2_ and *H*_peak_ suggest that a crossover may occur in the vicinity of 131.5–131.75 K. In this region the postive peak in Δ*S*_M_(*T*) begins to deviate from *H*_C,2_ determined from Δ*S*_M_(Δ*H*) (Figure S1 in Supplementary Information). At temperatures above this boundary (small black squares), thermal fluctuations compete with the magnetic field-induced commensuration that disorders the chiral ground state, and the metamagnetic crossover from linear CSL to highly nonlinear CSL eventually disappears.

In summary, a comprehensive phase diagram was constructed for the chiral helimagnet Cr_1/3_NbS_2_ by analyzing three magnetic field regimes. Critical exponents analysis at high magnetic field shows that the localized Cr^3+^ moments fall into the 3D Heisenberg universality class with exponents *β* = 0.3460 ± 0.040 *γ* = 1.344 ± 0.002, and confirms the second-order phase transition from the FFM to PM state at *T*_C_ = 130.78 K ± 0.044. In the field-polarized state, the ferromagnetic subsystem is decoupled from the DM interaction and reveals short-range isotropic interactions. Below ~1 kOe, the coherent long-range order of the CSL and CHM phase is set by the crystalline chirality. The magnetocaloric effect was used to calculate the magnetic entropy change, Δ*S*_M_(*T*), to map out the boundaries separating the CHM, CSL, and FFM regions of the phase diagram. An order-disorder critical temperature was defined at T* ~ 132.5 K, where the chiral phase exists above the Curie temperature, which agrees with the behavior shown theoretically in ref. 12. Using the condition to test universality of Δ*S*_M_(*T*), we find that failure of collapse of the rescaled Δ*S*_M_(*T*) for fields Δ*H* = 25 Oe–425 Oe indicates that a first-order transition likely occurs in the region Δ*T* = *T*_C_ − T* and is suppressed to second-order at higher applied magnetic field.

## Methods

Single crystals of Cr_1/3_NbS_2_ were grown with a chemical vapor transport method using I_2_ gas that has been reported elsewhere^20^. Magnetic measurements were performed using a Quantum Design Physical Property Measurement System (PPMS) with a Vibrating Sample Magnetometer (VSM) option. A warming protocol was adopted in which the sample was heated between each measurement to 200 K - well above *T*_C_ - to minimize any remanent effects and to account for possible irreversibility. Magnetic entropy change and critical exponents were calculated from isothermal magnetization versus magnetic field data measured up to 30 kOe and for temperature range 110–140 K. Temperature and field steps for the range 125 K ≤ *T*_C_ ≤ 133 K were measured in intervals of 0.25 K and 25 Oe, respectively. Magnetization vs. temperature was measured from 50–140 K for applied magnetic fields ranging from 0–2000 Oe.

## Electronic supplementary material

Supplementary Information
