# Supplementary material for: Critical Behavior and Macroscopic Phase Diagram of the Monoaxial Chiral Helimagnet Cr1/3NbS2
Source: Sci Rep. 2017 Jul 26;7:6545. doi: 10.1038/s41598-017-06728-5 (PMC5529428; doi:10.1038/s41598-017-06728-5)
Supplement: Supplementary file 1 — Supplementary Information [file 41598_2017_6728_MOESM1_ESM.pdf]

Critical Behavior and Macroscopic Phase Diagram of the Monoaxial Chiral Helimagnet $Cr_{1/3}NbS_2$

Supplementary Information

Eleanor M. Clements[1], Raja Das[1], Ling Li[2], Paula J. Lampen-Kelley[2], Manh-Huong Phan[1,*],
Veerle Keppens[2], David Mandrus[2], and Hariharan Srikanth[1,*]

[1]Department of Physics, University of South Florida, Tampa, FL 33620, USA

[2]Department of Materials Science and Engineering, University of Tennessee, Knoxville, Tennessee 37996, USA

*Corresponding authors: phanm@usf.edu and sharihar@usf.edu

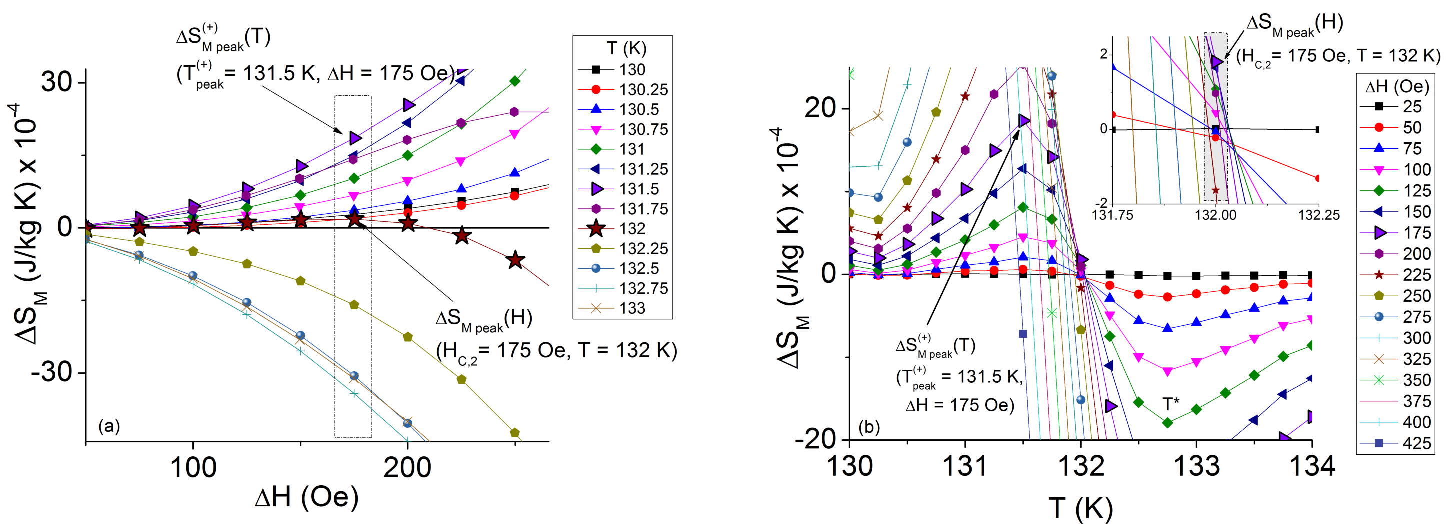

Figure S.1 $\Delta S_{\mathrm{M}}(\Delta H)$ and $\Delta S_{\mathrm{M}}(T)$ behavior in the region of the phase diagram represented in Figure 6(b) in the main text. (a) The boxed data, which lies on the iso-field line of 175 Oe, represents the $\Delta S_{\mathrm{M}}(T)$ curve for $\Delta H = 175$ Oe in (b), shown by the purple triangles. (b) An analogous scenario can be observed by comparing the boxed area in the inset with the data in red stars in (a). In Figure 6(b) it can be seen that for temperatures below the boundary marked by the positive peak in $\Delta S_{\mathrm{M}}(T)$, magnetic entropy is positive-increasing with both temperature and field. On the high temperature side of the positive peak in $\Delta S_{\mathrm{M}}(T)$, magnetic entropy change is positive-decreasing with temperature, but positive-increasing with field. Thus, at temperatures above the positive peak in $\Delta S_{\mathrm{M}}(T)$, thermal fluctuations begin to stabilize the chiral ground state against the disordering nature of the background magnetic field.
